# Characteristic of Perineural Invasion in Hilar Cholangiocarcinoma Based on Whole-Mount Histologic Large Sections of Liver

**DOI:** 10.3389/fonc.2022.855615

**Published:** 2022-03-08

**Authors:** Si-Yuan Wang, Nan Jiang, Jian-Ping Zeng, Shao-Qing Yu, Ying Xiao, Shuo Jin

**Affiliations:** ^1^Beijing Tsinghua Changgung Hospital, School of Clinical Medicine, Institute for Precision Medicine, Tsinghua University, Beijing, China; ^2^State Key Laboratory of Precision Measurement Technology and Instruments, Department of Precision Instrument, Tsinghua University, Beijing, China; ^3^Department of Pathology, Beijing Tsinghua Changgung Hospital, Beijing, China

**Keywords:** panoramic immunohistochemistry, whole-mount histologic large sections, tumor biological behavior, pathological digital imaging, pathological methodology

## Abstract

**Background & Objective:**

Perineural invasion is an important biological feature of hilar cholangiocarcinoma (HCCA). We developed a whole-mount histologic large sections (WHLS) of the liver to evaluate peripheral nerve invasion (PNI) of HCCA.

**Methods:**

Using sampling, fixation, dehydration, embedding, sectioning, hematoxylin and eosin (H&E) and immunohistochemical (IHC) staining, and scanning, the characteristics of intrahepatic and extrahepatic PNI in 20 patients with Bismuth type III and type IV HCCA were analyzed with WHLS. Correlation between the characteristics of nerve invasion and tumor size, vascular invasion (artery, portal vein), degree of differentiation, microvascular invasion (MVI), carbohydrate antigen19-9 (CA19-9), and differentiation degree of HCCA was statistically evaluated.

**Results:**

The WHLS of the liver was successfully established, which enabled us to observe intrahepatic and extrahepatic distribution of HCCA and whether surrounding tissues including nervous, blood, and lymph vessels were infiltrated. Extrahepatic and intrahepatic PNI were identified in 20 (100%) patients and 1 (5.0%) patient, respectively. Vessel density decreased in most invaded nerves presented by CD-34, which correlated with 100% of poorly differentiated and 83% of moderately differentiated tumors (P<0.008).

**Conclusion:**

This study established a WHLS of the liver that can be used for clinical diagnosis and research, and confirmed that extrahepatic PNI is prevalent, but intrahepatic nerve invasion is rare and does not accompany the invasion scope of bile ducts in types III and IV HCCA. In addition, moderately and poorly differentiated malignant tumors are more prone to PNI, independent of blood supply.

## 1 Introduction

Hilar cholangiocarcinoma (HCCA) is the most common type of cholangiocarcinoma, and is characterized by high morbidity, high mortality rates, and poor prognosis (1–5). According to previous studies, the proportion of peripheral nerve invasion (PNI) in HCCA is around 38.8%-84.5% (6–10), and the overall survival rate of patients with PNI is significantly shorter than of those without PNI (11–13). In types III and IV HCCA, the invasion scope in the perihilar region is significantly larger than that of types I and II, and the tumor invades further along the intrahepatic bile duct. However, whether there is a difference between intrahepatic and extrahepatic PNI has not been clearly determined. Owing to limitations of the study area and due to tissue discontinuity, traditional pathological methods have not been able to provide solutions to this problem (14–16). Therefore, a whole-mount histologic large sections (WHLS) technology for hepatectomy specimens larger than 10 cm × 10 cm is urgently needed to panoramically analyze the characteristics of nerve invasion in HCCA.

WHLS technology was first introduced in 1994 for the diagnosis and staging of breast, prostate, endometrial, and lung cancers as well as brain tumors (15, 17–19). However, no systematic studies on hepatic WHLS larger than 5cm have been reported.

We have developed a WHLS system that includes dehydration, tissue embedding, sectioning, hematoxylin and eosin (H&E), immunohistochemical (IHC) staining and digital scanning for hepatectomy specimens larger than 10 × 10cm. Through this system, we can observe the characteristics of intrahepatic and extrahepatic PNI in Bismuth III and IV types of HCCA. Besides, WHLS can display the entire tumor, its surrounding tissues, the intrahepatic lesions, the incision margin, and the surrounding microenvironment.6

## 2 Materials and Methods

Twenty patients were diagnosed with Bismuth types III and IV HCCA and underwent hemihepatectomy or trisegmenthepatectomy from July 2018 to February 2021. The preparation and staining methods of WHLS were as follows. IHC staining of S100, CD-34, and D2-40 was performed to visualize the nerve, lymph vessels, and blood vessels. Clinical and pathological information including tumor size, vascular invasion, degree of differentiation, microvascular invasion (MVI), and carbohydrate antigen19-9 (CA19-9) were recorded and analyzed. We used the broad definition of PNI as tumor cell invasion in, around, and through the nerves (5).

### 2.1 Establishment of Hepatic WHLS

#### 2.1.1 Equipment and Apparatus Used to Prepare WHLS

The customized glass slide dimensions were 127 mm × 102 mm × 1.1 mm and the customized cover glass dimensions were 120 mm × 100 mm × 0.13 mm. The study area of WHLS was approximately 60 times larger than that of traditional pathological sections. Other equipment included a Leica ASP300S tissue processor (Leica Microsystems, Wetzlar, Germany), a Sakura IVS-410 microtome (Sakura Finetek Japan Co. Ltd., Tokyo, Japan), and a Sakura Tissue-Tek TEC5 embedding machine (Sakura Finetek Japan Co. Ltd.).

#### 2.1.2 First and Second Fixation Stages

In the first fixation stage, the resected liver specimens were dissected and photographed. Ten percent (v/v) neutral formalin fixation solution was injected into the liver specimens including the hepatic arteries, portal veins, and bile duct systems to ensure complete tissue fixation. In the second fixation stage, the specimens were immersed in 10-fold volumes of 10% (v/v) formalin for 24 h.

#### 2.1.3 Tissue Sampling of WHLS

Specimens were taken along a plane parallel to the axial abdominal enhanced CT (**Supplement Figure 1A**). Continuous cross-sections, 8–10 mm in thickness, were sampled. The starting sites from the distal end of the tumor until the intrahepatic tumor disappeared.

#### 2.1.4 Third Fixation Stage

HCCA cross-sections, 8–10 mm in thickness, were sampled for the second time. To ensure the smoothness of the specimens, the thickness of each section was controlled within 4–5 mm using the embedding frame as a guide (**Supplement Figure 1B**). The modified tissue was placed in a tenfold volume of 10% (v/v) neutral formalin, re-fixed for several days.

#### 2.1.5 Dehydration

This procedure is a critical step in WHLS preparation. Here, a suitable dehydration procedure was established after several preliminary experiments. The sections were placed in a Leica ASP300S dehydrator (Leica Microsystems) under normal pressure. The dehydration procedure is shown in **Supplement Table 1**.

#### 2.1.6 Tissue Embedding and Paraffin Impregnation

For WHLS, a glass culture dish approximately 30 cm in diameter, was used for paraffin impregnation and embedding (**Supplement Figure 1C**). Dental forceps were then used to adjust and press the tissue and ensure that it was neither curled nor deformed. **Supplement Figure 1D** shows the properly prepared paraffin-embedded tissues.

#### 2.1.7 Tissue Sectioning

The Sakura IVS-410 microtome and Sakura disposable 18-cm custom blade were used for slicing. Serial sections were obtained at a distance of 2 mm from the beginning of the common hepatic duct tumor until the proximal liver tumor disappeared along a plane parallel to the axial abdominal enhanced CT. The temperature of the Leica spreading sink (Leica Microsystems) was set to 39°C while the temperature of the Leica baking table (Leica Microsystems) was set to 75°C. High-flow humidification was critical during sectioning.

#### 2.1.8 H&E Staining

The steps of H&E staining are listed in **Supplement Table 2**.

#### 2.1.9 IHC Staining

After deparaffinization and antigen retrieval, all sections were incubated with primary anti-S-100 antibody, anti-CD34 antibody, and anti-D2-40 antibody (Abcam, USA) overnight at room temperature. After washing with PBS-T, all sections were incubated with secondary antibody (Abcam, USA) for 60 min at room temperature. Finally, xylene was used for transparency, and neutral gum was used to seal the tablets.

#### 2.1.10 Digital Scanning and Interpretation of WHLS Features

Tissue sections were digitally scanned with the Olympus VS200 panoramic digital scanner (Olympus Corp., Tokyo, Japan). Characteristics of the scanned WHLS were interpreted with Olyvia software (Olympus Corp.). This program freely expanded and contracted WHLS at a maximum magnification ≥ 400×. All features of the HCCA were interpreted by three senior members of the Department of Pathology. In case there was a lack of consensus among the three pathologists, a fourth was consulted to interpret the features.

### 2.2 Statistical Analysis

Results are expressed as means ± standard deviation. Statistical analyses were carried out using the chi square test t-test, and Mann–Whitney U test by SPSS v25.0. A p value of less than 0.05 was considered statistically significant.

## 3 Results

The clinicopathological characteristics of the 20 cases are shown in **Table 1**. According to the Bismuth-Corlette classification, 12 patients were type III (60%) and 8 patients were type IV (40%). Pathological analysis shows that there were 11 infiltrating (55%), 3 nodular (15%), and 6 sclerotic (30%) types. A total of 15 (75%) patients underwent hemihepatectomy and 5 (25%) patients underwent trisegment hepatectomy.

**Table 1:** Demographic and preoperative data.

	No. of patients (n = 20)
**Age (years)**	66.1 (44-79)
**Sex ratio (M: F)**	9: 11
**Bismuth type**	
III A	6 (30%)
III B	6 (30%)
IV	8 (40%)
**Surgical operation**	
Right hemitectectomy	8 (40%)
Left hemitectectomy	7 (35%)
Right trisegment hepatectectomy	2 (10%)
Left trisegment hepatectectomy	3 (15%)
**Pathological type**	
Infiltrating	11 (55%)
Sclerotic	6 (30%)
Nodular	3 (15%)

### 3.1 Display of WHLS in HCCA

**Figure 1:**
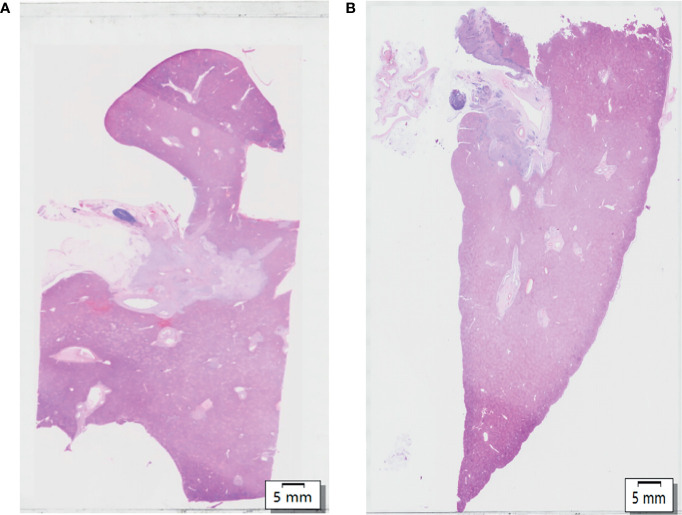
**(A, B)** Hematoxylin and eosin (H&E) of whole-mount histologic large sections (WHLS) in Hilar cholangiocarcinoma (HCCA). Scale bar, 5 mm.

The WHLS of HCCA in **Figures 1A, B** included the caudate lobe and the hilum. Moreover, adipose, nerve tissue, vessels, and lymph nodes in the hepatic hilar region could also be observed.

### 3.2 Display of the Caudate Lobe and Hilum in HCCA

**Figure 2:**
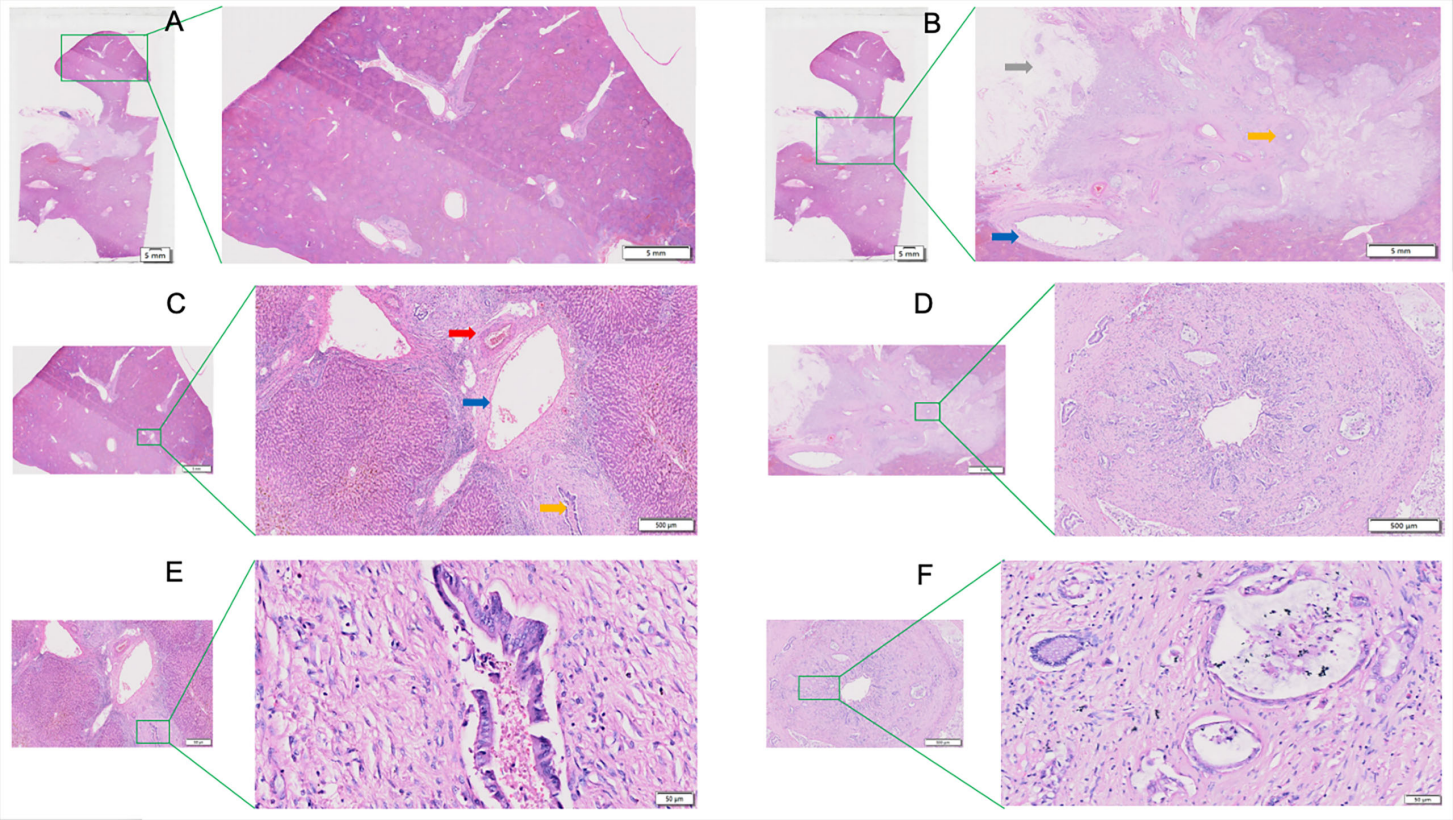
View of the caudate lobe by WHLS in HCCA. **(A)** Large portal areas visible within caudate lobes. Scale bar, 5 mm. **(B)** Tissues in the hilar region including portal vein (blue arrow), bile duct (yellow arrow), adipose tissue(grey arrow). Scale bar, 5 mm. **(C)** Arterioles (red arrow), portal vein (blue arrow), and bile ducts (yellow arrow) in caudate lobular portal area. Scale bar, 500um. **(D)** Cancerous bile duct. Scale bar, 500 µm. **(E)** Bile ducts in caudate lobular portal area. Scale bar, 50 µm. **(F)** Adenocarcinoma in cancerous bile duct. Scale bar, 50 µm.

The caudate lobe region and hilar region shown in **Figure 1A** were enlarged to obtain **Figures 2A–F**. The arrangements of the hepatocytes, Glisson system, and other features of the tissues in the caudate lobe and hilum were clearly visible. Furthermore, the cancerous bile duct and changes in the surrounding environment could be comprehensively mapped.

### 3.3 Display of Hepatic Margin and Peripheral Portal Area

**Figure 3:**
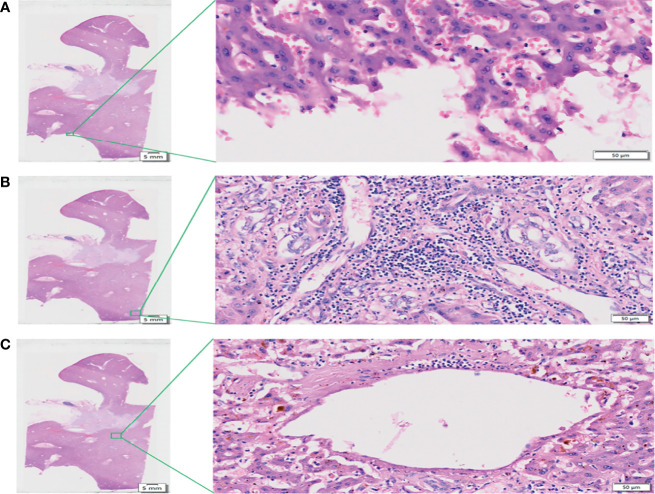
View of the liver incision margin, distal peripheral portal area, and internal hepatic parenchyma of WHLS in HCCA. **(A)** Local liver incision margin. Scale bar, 50 µm. **(B)** Distal peripheral portal area. Scale bar, 50 µm. **(C)** Internal hepatic parenchyma. Scale bar, 50 µm.

**Figure 3A** is an enlargement of the local incision margin shown in **Figure 1A** and clearly demonstrates hepatocyte morphology and hepatocyte cord structure. **Figure 3B** is an enlargement of the distal peripheral portal area and clearly demonstrates the morphological characteristics of the bile ducts and their relationship with neighboring tissues. **Figure 3C** shows the internal hepatic parenchyma and the central vein and the surrounding hepatocytes are clearly visible.

### 3.4 Display of Intrahepatic and Extrahepatic Nerves, Blood Vessels, and Lymph Vessels

**Figure 4:**
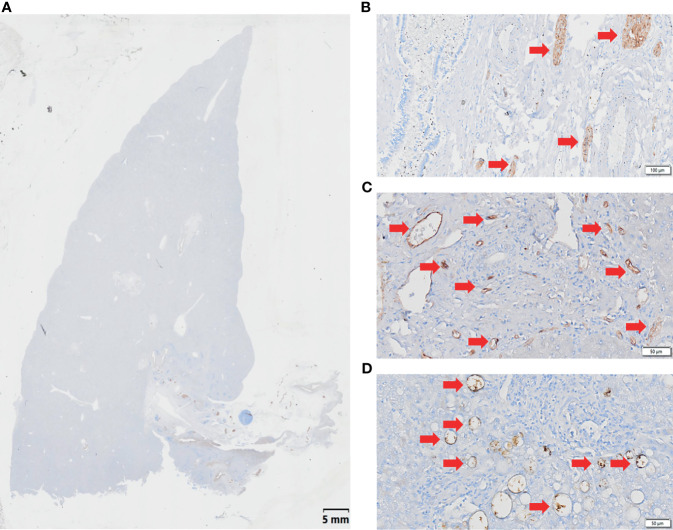
Immunohistochemical (IHC) staining of WHLS in HCCA. **(A)** IHC staining. Scale bar, 5 mm. **(B)** S-100 staining demonstrates nerve distribution in the liver. The red arrows show nerve tissue. Scale bar, 50 µm. **(C)** CD34 staining demonstrates vascular distribution in the liver. The red arrows show blood vessels. Scale bar, 100 µm. **(D)** D2-40 staining demonstrates lymphatic distribution in the liver. The red arrows show lymphatic vessels. Scale bar, 50 µm.

Through S-100, CD-34, and D2-40 IHC staining, combined with HE staining results, the distribution of intrahepatic and extrahepatic nerves, vessels, and lymph-vessels was clearly displayed (**Figures 4A–D**).

### 3.5 Characteristics and Risk Factors of PNI

**Figure 5:**
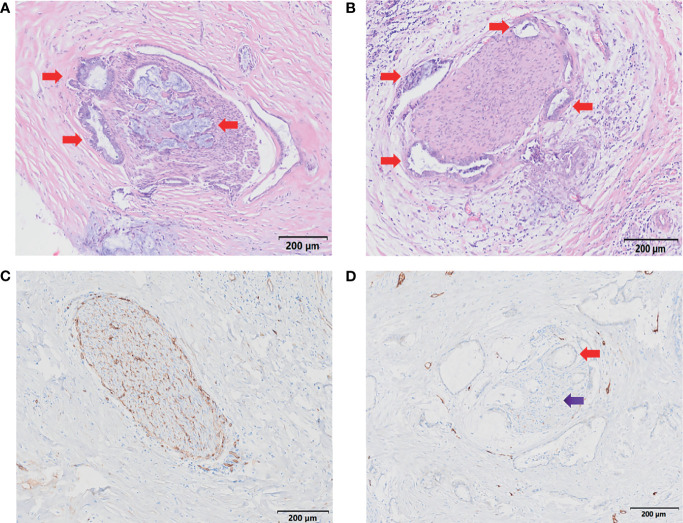
Characteristics of PNI. **(A, B)** H&E staining demonstrates nerve invasion (red arrows), including both perineural invasion **(A, B)** and intraneural invasion **(A)**. Scale bar, 200 µm. **(C)** Distribution of blood supply to normal nerves (S-100 staining). Scale bar, 200 µm. **(D)** Blood supply distribution of PNI (S-100 staining); the red arrow shows tumor invasion of the nerve, and the purple arrow shows decreased blood supply to the nerve. Scale bar, 200 µm.

After H&E and IHC staining, the intrahepatic and extrahepatic nerves and vessels of excised liver specimens were clearly displayed. Among the 20 patients, the rate of PNI was 100%. For the 20 treated cases, extrahepatic and intrahepatic PNI were identified in 20 (100%) patients and 1 (5.0%) patient, respectively (**Figures 5A, B** and **Table 2**). The pathological type in the patients with intrahepatic PNI was infiltrating (Bismuth IIIB). When the nerve was invaded by tumors, vessel density decreased in 16 patients (80%) and showed no change in 4 patients (20%) as shown by CD34 IHC staining (**Figures 5C, D**). The decreased vessel density in PNI was highly statistically correlated with the degree of tumor differentiation, which occurred in 100% of poorly differentiated and 83% of moderately differentiated tumors (P<0.008, **Table 3**). Moreover, our study confirmed that vessel density decrease in PNI was not statistically associated with tumor size, Bismuth type, vascular invasion (including hepatic artery and portal vein), CA-199, MVI, or Ki-67.

**Table 2:** Characteristics of PNI.

	Intrahepatic PNI	Extrahepatic PNI	CD34
**1**	Negative	Positive	Decrease
**2**	Negative	Positive	Decrease
**3**	Negative	Positive	Decrease
**4**	Negative	Positive	Decrease
**5**	Negative	Positive	Decrease
**6**	Negative	Positive	Normal
**7**	Negative	Positive	Normal
**8**	Negative	Positive	Decrease
**9**	Negative	Positive	Decrease
**10**	Negative	Positive	Decrease
**11**	Negative	Positive	Decrease
**12**	Negative	Positive	Decrease
**13**	Positive	Positive	Decrease
**14**	Negative	Positive	Decrease
**15**	Negative	Positive	Decrease
**16**	Negative	Positive	Normal
**17**	Negative	Positive	Decrease
**18**	Negative	Positive	Normal
**19**	Negative	Positive	Decrease
**20**	Negative	Positive	Decrease

**Table 3:** Risk factors of PNI.

	Decrease of blood supply	Normal blood supply	P value
**Pathological Type**			0.487
Nodular	3 (18.8%)	0 (0.0%)	
Infiltrating	9 (56.3%)	2 (50%)	
Sclerotic	4 (25.0%)	2 (50%)	
**Bismuth type**			0.255*
III	11 (68.8%)	1 (25%)	
IV	5 (31.3%)	3 (75%)	
**Vascular invasion (A)**			0.619*
positive	7 (87.5%)	9 (25%)	
negative	1 (12.5%)	3 (75%)	
**Vascular invasion (P)**			0.582*
positive	9 (90.0%)	7 (70%)	
negative	1 (10.0%)	3 (30%)	
**Differentiation**			0.008
low	6 (37.5%)	0 (0.0%)	
**moderate**	10 (62.5%)	2 (50.0%)	
high	0 (0.0%)	2 (50.0%)	
**MVI**			1.000*
negative	13 (81.3%)	4 (100.0%)	
positive	3 (18.8%)	0 (0.0%)	
**CA-199**	565.80 (107.85, 1178.80)	1178.80 (536.61, 3194.27)	0.104
**Ki-67**	20.0% (15.0%, 35.0%)	20.0% (18.5%, 35.0%)	0.739

*Fisher exact test; A, hepatic artery; P, portal vein; MVI, microvascular invasion; CA19-9, carbohydrate antigen 19-9.

## 4 Discussion

In this study, we developed a WHLS that clarified the incidence of PNI in types III and IV HCCA, and the characteristics of the blood supply of the PNI inside the liver were further clarified. Moreover, the panoramic digital pathology system provided a relatively more complete qualitative diagnosis after exploring the scope of invasion and the biological behavior of the tumor.

### 4.1 Establishment of WHLS

A three-stage tissue fixation scheme was adopted in this study. The first involved perfusion fixation. The perfusion liquid penetrated as far as possible into the liver parenchyma and prevented the sloughing of the intrahepatic duct epithelium. The second stage aimed to increased specimen hardness and enabled the application of the third fixation stage. In the third stage, specimens were further processed into thin lamellae, approximately 4-5 mm thick, facilitating an even more profound fixation of the liver parenchyma to prevent autolysis.

To the best of our knowledge, no prior studies have reported on the systemic dehydration of liver tissue specimens > 10 cm in size (14, 17, 19). Dehydration for large liver tissue pathological investigations should endeavor to prevent wrinkling, maintain tissue hardness, and facilitate sectioning. In this study, we developed a series of dehydration procedures suitable for whole-mount liver that fully dehydrated the tissue samples and minimized shrinkage. Prolonged tissue dehydration at low alcohol concentrations (70%) effectively increases dehydration efficiency within the liver parenchyma. The amount of time in which tissues are soaked in high alcohol concentrations is minimized. In this way, tissue shrinkage is avoided, and the tissue surface remains smooth.

In this study, we performed H&E staining and IHC staining manually after optimizing it through repeated preliminary tests. **Supplement Table 2** shows that the H&E staining time for large sections was approximately 84-86 min. Adenocarcinoma, the surrounding liver tissue, and its nuclear morphology were clearly visible in the pathological liver sections stained by this technique. In addition, the morphology of all accessory arteries, blood vessels, and lymphatic and nerve tissues could be plainly seen.

### 4.2 PNI Characteristics

Previous studies have reported that there are great differences in the probability of nerve invasion in HCCA (6, 12, 20–22). Moreover, the characteristics of nerve invasion and whether it is accompanied by intrahepatic bile duct invasion in types III and IV of HCCA are not clear. Our study established a WHLS of the liver to show that the rate of PNI was prevalent (100%) in Bismuth types III and IV HCCA. This may indicate that, compared with types I and II HCCA, the biological behavior of extrahepatic PNI in types III and IV HCCA was increased owing to the large infiltration range in the perihilar region. We have further confirmed the pattern of the PNI using WHLS, suggesting that PNI was more common in the extrahepatic (100%) than in the intrahepatic (5.0%) area. This result confirmed that PNI in types III and IV HCCA was not accompanied by intrahepatic bile duct invasion. A previous study demonstrated that when tumor cells invade nerve fibers, the number of blood vessels in the peripheral tissues increase (5, 23, 24). By using WHLS, our study revealed that the blood supply was rich and well distributed in normal nerves. However, there was a decrease in the density of vessels in PNI in most cases of III and IV HCCA, indicating that these two types of HCCA may not rely on blood supply for invading nerves. We also found that this biological behavior was more common in tumors with low (100%) and moderate (83%) differentiation (P<0.008). These results suggest that the moderately and poorly differentiated malignant tumors are more prone to PNI independent of blood supply.

In conclusion, this study established a WHLS of the liver that can be used for clinical diagnosis and research, and confirmed that extrahepatic PNI is prevalent in types III and IV HCCA, but intrahepatic PNI is rare and does not accompany the invasion scope of the bile duct. The decrease in vessel density in PNI was closely related to tumor differentiation. The more undifferentiated the HCCA, the more likely will be the biological behavior of PNI independent of blood supply to appear.

## Data Availability Statement

The raw data supporting the conclusions of this article will be made available by the authors, without undue reservation.

## Ethics Statement

The studies involving human participants were reviewed and approved by Beijing Tsinghua Changgung Hospital Ethics Committee. Written informed consent for participation was not required for this study in accordance with the national legislation and the institutional requirements.

## Author Contributions

Conceptualization, S-YW, NJ, and SJ. Methodology, S-YW. Software, S-YW and S-QY. Validation, NJ, J-PZ, and SJ. Formal analysis, S-YW. Investigation, S-YW. Resources, SJ. Data curation, YX. Writing—original draft preparation, NJ and S-YW. Writing—review and editing, SJ. Visualization, S-QY. Supervision, SJ. Project administration, J-PZ. All authors have read and agreed to the published version of the manuscript.

## Funding

National Natural Science Foundation of China (No. 82000484, No. 81930119, No. 82090050, No. 82090053). Research and Cultivation Program of Beijing Municipal Hospital (No. PX2018038).

## Conflict of Interest

The authors declare that the research was conducted in the absence of any commercial or financial relationships that could be construed as a potential conflict of interest.

## Publisher’s Note

All claims expressed in this article are solely those of the authors and do not necessarily represent those of their affiliated organizations, or those of the publisher, the editors and the reviewers. Any product that may be evaluated in this article, or claim that may be made by its manufacturer, is not guaranteed or endorsed by the publisher.

## Supplementary Material

Supplementary Figure 1 Histologic tissue sampling, second tissue sampling, and paraffin embedding of hilar cholangiocarcinoma (HCCA) pathological tissue. **(A)** Whole-mount histologic large sections (WHLS) taken along the axial CT direction. **(B)** Pathological tissue sampling after second fixation. **(C)** Paraffin embedding of HCCA pathological tissue. **(D)** HCCA pathological tissue embedding being prepared.

## Abbreviations

HCCA, hilar cholangiocarcinoma; PNI, peripheral nerve invasion; H&E, hematoxylin-eosin staining; IHC, immunohistochemical staining; WHLS, whole-mount histologic large sections.

## References

[1] Razumilava N Gores GJ. Cholangiocarcinoma. Lancet (2014) 383(9935):2168–79. doi: 10.1016/s0140-6736(13)61903-0 PMC4069226 24581682

[2] Valle JW Kelley RK Nervi B Oh D-Y Zhu AX. Biliary Tract Cancer. Lancet (2021) 397(10272):428–44. doi: 10.1016/s0140-6736(21)00153-7 33516341

[3] Poruk KE Pawlik TM Weiss MJ. Perioperative Management of Hilar Cholangiocarcinoma. J Gastrointest Surg (2015) 19(10):1889–99. doi: 10.1007/s11605-015-2854-8 PMC4858933 26022776

[4] Lewis HL Rahnemai-Azar AA Dillhoff M Schmidt CR Pawlik TM. Current Management of Perihilar Cholangiocarcinoma and Future Perspectives. Chirurgia (Bucur) (2017) 112(3):193–207. doi: 10.21614/chirurgia.112.3.193 28675356

[5] Liebig C Ayala G Wilks JA Berger DH Albo D. Perineural Invasion in Cancer: A Review of the Literature. Cancer (2009) 115(15):3379–91. doi: 10.1002/cncr.24396 19484787

[6] Hu HJ Jin YW Shrestha A Ma WJ Wang JK Liu F. Predictive Factors of Early Recurrence After R0 Resection of Hilar Cholangiocarcinoma: A Single Institution Experience in China. Cancer Med (2019) 8(4):1567–75. doi: 10.1002/cam4.2052 PMC6488134 30868740

[7] Seyama Y Kubota K Sano K Noie T Takayama T Kosuge T. Long-Term Outcome of Extended Hemihepatectomy for Hilar Bile Duct Cancer With No Mortality and High Survival Rate. Ann Surg (2003) 238(1):73–83. doi: 10.1097/01.Sla.0000074960.55004.72 12832968 PMC1422671

[8] Robles R Figueras J Turrión VS Margarit C Moya A Varo E. Spanish Experience in Liver Transplantation for Hilar and Peripheral Cholangiocarcinoma. Ann Surg (2004) 239(2):265–71. doi: 10.1097/01.sla.0000108702.45715.81 PMC1356221 14745336

[9] Su CH Tsay SH Wu CC Shyr YM King KL Lee CH. Factors Influencing Postoperative Morbidity, Mortality, and Survival After Resection for Hilar Cholangiocarcinoma. Ann Surg (1996) 223: (4):384–94. doi: 10.1097/00000658-199604000-00007 PMC1235134 8633917

[10] Yamaguchi K Chijiiwa K Saiki S Shimizu S Tanaka M. Carcinoma of the Extrahepatic Bile Duct: Mode of Spread and its Prognostic Implications. Hepatogastroenterology (1997) 44(17):1256–61. doi: 10.1002/(SICI)1097-0347(199709)19:6<535::AID-HED11>3.0.CO;2-4 9356836

[11] Nimura Y Kamiya J Kondo S Nagino M Uesaka K Oda K. Aggressive Preoperative Management and Extended Surgery for Hilar Cholangiocar Cinoma: Nagoya Experience. J Hepato Biliary Pancreatic Surg (2000) 7(2):155–62. doi: 10.1007/s005340050170 10982608

[12] Tan X Sivakumar S Bednarsch J Wiltberger G Heij LR. Nerve Fibers in the Tumor Microenvironment in Neurotropic Cancer—Pancreatic Cancer and Cholangiocarcinoma. Oncogene (2020) 40:899–908. doi: 10.1038/s41388-020-01578-4 33288884 PMC7862068

[13] Aloe L Rocco ML Balzamino BO Micera A. Nerve Growth Factor: Role in Growth, Differentiation and Controlling Cancer Cell Development. J Exp Clin Cancer Res (2016) 35(1):116–23. doi: 10.1186/s13046-016-0395-y PMC4955168 27439311

[14] Mukhopadhyay S Feldman MD Abels E Ashfaq R Beltaifa S Cacciabeve NG. Whole Slide Imaging Versus Microscopy for Primary Diagnosis in Surgical Pathology. Am J Surg Pathol (2017) 00(00):1–14. doi: 10.1097/PAS.0000000000000948 PMC5737464 28961557

[15] Lazutkin AA Shuvaev SA Barykina NV. Click Histochemistry for Whole-Mount Staining of Brain Structures. MethodsX (2019) 6:1986–91. doi: 10.1016/j.mex.2019.09.011 PMC6812327 31667095

[16] Volynskaya Z Evans AJ Asa SL. Clinical Applications of Whole-Slide Imaging in Anatomic Pathology. Adv Anat Pathol (2017) 24(4):215–21. doi: 10.1097/PAP.0000000000000153 28590953

[17] Cimadamore A Cheng L Lopez-Beltran A Mazzucchelli R Luciano R Scarpelli M. Added Clinical Value of Whole-Mount Histopathology of Radical Prostatectomy Specimens: A Collaborative Review. Eur Urol Oncol (2021) 4(4):558–69. doi: 10.1016/j.euo.2020.08.003 32883645

[18] Ebel JJ Shabsigh A Sharp DS Zynger DL. Whole-Mount Evaluation of Penectomies for Penile Cancer: Feasibility, Cost and Comparison to Routine Sectioning. Histopathology (2013) 63(1):64–73. doi: 10.1111/his.12149 23738630

[19] Clarke GM Eidt S Sun L Mawdsley G Zubovits JT Yaffe MJ. Whole-Specimen Histopathology: A Method to Produce Whole-Mount Breast Serial Sections for 3-D Digital Histopathology Imaging. Histopathology (2007) 50(2):232–42. doi: 10.1111/j.1365-2559.2006.02561.x 17222252

[20] Kovalenko YA Zharikov YO Kukeev IA Vishnevsky VA Chzhao AV. Predictors of Outcomes in Surgery for Hilar Cholangiocarcinoma. Khirurgiia (Mosk) (2018) 10):5–11. doi: 10.17116/hirurgia20181015 30531729

[21] Chen S-H Zhang B-Y Zhou B Zhu C-Z Sun L-Q. Perineural Invasion of Cancer: A Complex Crosstalk Between Cells and Molecules in the Perineural Niche. Am J Cancer Res (2019) 9(1):1–21. 30755808 PMC6356921

[22] Mansfield SD Barakat O Charnley RM Jaques BC O’Suilleabhain CB Atherton PJ. Management of Hilar Cholangiocarcinoma in the North of England: Pathology, Treatment, and Outcome. J World J Gastroenterol (2005) 11(48):7625–30. doi: 10.3748/wjg.v11.i48.7625 PMC4723393 16437689

[23] Li CG Zhou ZP Tan XL Zhao ZM. Perineural Invasion of Hilar Cholangiocarcinoma in Chinese Population: One Center’s Experience. World J Gastrointest Oncol (2020) 12(4):457–66. doi: 10.4251/wjgo.v12.i4.457 PMC7191337 32368323

[24] Shen FZ Zhang BY Feng YJ Jia ZX An B Liu CC. Current Research in Perineural Invasion of Cholangiocarcinoma. J Exp Clin Cancer Res (2010) 29(1):1–7. doi: 10.1186/1756-9966-29-24 20219134 PMC2851676

