# Supplementary material for: Characteristic of Perineural Invasion in Hilar Cholangiocarcinoma Based on Whole-Mount Histologic Large Sections of Liver
Source: Front Oncol. 2022 Mar 8;12:855615. doi: 10.3389/fonc.2022.855615 (PMC8957852; doi:10.3389/fonc.2022.855615)
Supplement: Supplementary file 1 [file Table_1.docx]

**Supplement Table 1. Dehydration procedure**

| Procedure | Reagent | Time | Temperature |
| --- | --- | --- | --- |
| 1 | 10% formalin | 2 h | 35°C |
| 2 | 70% Ethanol | 3 h | 35°C |
| 3 | 80% Ethanol | 2 h | 35°C |
| 4 | 95% Ethanol | 1.5 h | 35°C |
| 5 | 95% Ethanol | 1 h | 35°C |
| 6 | 95% Ethanol | 1 h | 35°C |
| 7 | 100% Ethanol | 1 h | 35°C |
| 8 | 100% Ethanol | 1 h | 35°C |
| 9 | Xylene | 2 h | 35°C |
| 10 | Xylene | 2 h | 35°C |
| 11 | Paraffin | 1 h | 60°C |
| 12 | Paraffin | 1 h | 60°C |
| 13 | Paraffin | 1 h | 60°C |
| 14 | Paraffin | 1 h | 60°C |
| Time |  | 20.5 h |  |
