# Supplementary material for: Characteristic of Perineural Invasion in Hilar Cholangiocarcinoma Based on Whole-Mount Histologic Large Sections of Liver
Source: Front Oncol. 2022 Mar 8;12:855615. doi: 10.3389/fonc.2022.855615 (PMC8957852; doi:10.3389/fonc.2022.855615)
Supplement: Supplementary file 2 [file Table_2.docx]

**Supplement Table 2. H&E staining procedure**

| Procedure | Reagent | Time |
| --- | --- | --- |
| 1 | Xylene (Ⅰ) | 10 min |
| 2 | Xylene ( II ) | 10 min |
| 3 | Xylene ( III ) | 5 min |
| 4 | 100% Ethanol (Ⅰ) | 5 min |
| 5 | 100% Ethanol ( II ) | 5 min |
| 6 | 95% Ethanol | 5 min |
| 7 | 80% Ethanol | 5 min |
| 8 | Wash | 2 min |
| 9 | Distilled water | 2 min |
| 10 | Hematoxylin staining | 10 min |
| 11 | Wash | 3 min |
| 12 | 1% Hydrochloric acid ethanol | 30 s |
| 13 | Wash | 1 min |
| 14 | 1% Ammonia back to blue | 30 s |
| 15 | Wash | 1 min |
| 16 | 0.5% Eosin liquid staining (water-based eosin staining) | 1-3 min |
| 17 | Wash | 1 min |
| 18 | 80% Ethanol | 10 s |
| 19 | 95% Ethanol | 1 min |
| 20 | Absolute ethyl alcohol ( I ) | 5 min |
| 21 | Absolute ethyl alcohol ( II ) | 5 min |
| 22 | Xylene (Ⅰ) | 3 min |
| 23 | Xylene ( II ) | 3 min |
| Time |  | 84-86min |
