# Supplementary figures and images for: Characteristic of Perineural Invasion in Hilar Cholangiocarcinoma Based on Whole-Mount Histologic Large Sections of Liver
Source: Front Oncol. 2022 Mar 8;12:855615. doi: 10.3389/fonc.2022.855615 (PMC8957852; doi:10.3389/fonc.2022.855615)

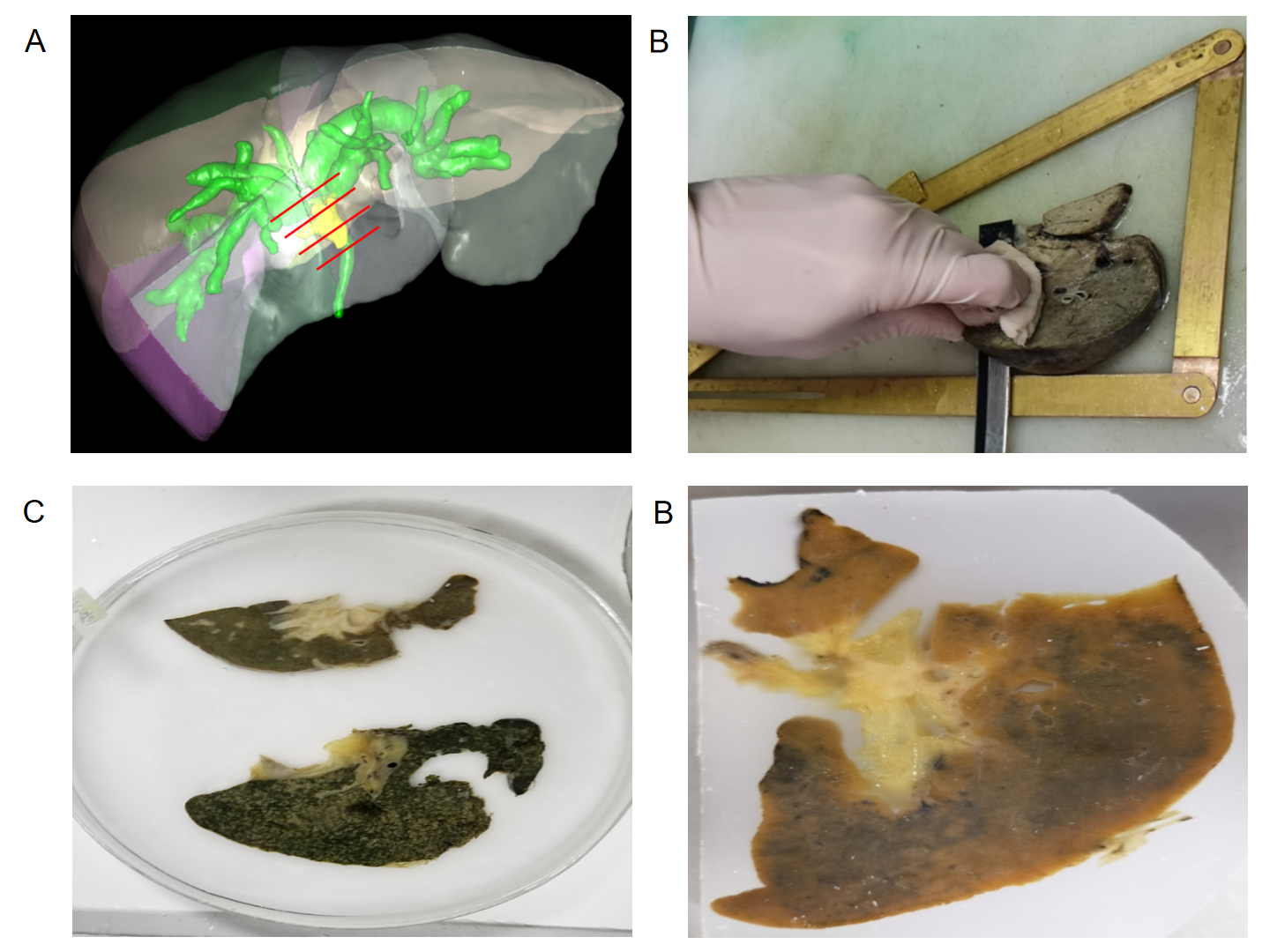

Supplement: Supplementary Figure 1 — Histologic tissue sampling, second tissue sampling, and paraffin embedding of hilar cholangiocarcinoma (HCCA) pathological tissue. (A) Whole-mount histologic large sections (WHLS) taken along the axial CT direction. (B) Pathological tissue sampling after second fixation. (C) Paraffin embedding of HCCA pathological tissue. (D) HCCA pathological tissue embedding being prepared. [file Image_1.tif]
